# Supplementary material for: Molecular Docking of Lac_CB10: Highlighting the Great Potential for Bioremediation of Recalcitrant Chemical Compounds by One Predicted Bacteroidetes CopA-Laccase
Source: Int J Mol Sci. 2023 Jun 6;24(12):9785. doi: 10.3390/ijms24129785 (PMC10298561; doi:10.3390/ijms24129785)
Supplement: Supplementary file 1 [file ijms-24-09785-s001.zip › ijms-2418478-supplementary.pdf]

Supplementary Material

Table S1. Identity analysis of Lac_CB10 through NCBI's BLASTp tool

Database colection	Organism	Accession	Per Ident/Query Cover
nr	*Chitinophaga jiangningensis*	WP_073081050.1	96.16%/100%
pat	Patente US9290773	APN18218.1	50%/64%
swissprot	Full Copper resistence protein A homolog (CopA)	Q.47452.1	33.16%/70%
PDB	*Botrytis aclada*	3V9E_A	33.21%/46%

Table S2. Physicochemical properties of Lac_CB10 predicted in Protparam

	Lac_CB10	
**Number of amino acids**	728	
**Theoretical ponto isoelétrico (pI)**	6.51	Condição da solução em que a proteína produz a mesma quantidade de cargas negativas e positivas, levando a uma carga final igual a zero [43].
**Molecular weight**	83292.19 KD	
**Instability index**	32.39	Índices de instabilidade (II) menores que 40 indicam uma proteína estável, enquanto valores acima de 40 tendem a representar instabilidade da proteína
**Aliphatic index**	72.13	O índice alifático representa o volume predito da proteína que é ocupado por aminoácidos alifáticos, como alanina. Um índice de valor alto (entre 42,08 e 90,68) indica que a proteína é estável em uma ampla faixa de temperaturas, ou seja, quanto mais o valor se aproximar de 90, mais termoestável será a proteína [43].

**Extinction coefficients**	1251.40	Coeficiente de extinção que é 125,140 M-1 cm-1, se refere à quantidade de luz que pode ser absorvida pela enzima em 280nm. É importante ter uma estimativa deste valor para se quantificar uma enzima pura pelo método de espectrofotometria [41].
**GRAVY**	-0.383	O parâmetro GRAVY indica média geral de hidrofobicidade (Grand Average of Hydropathy), sendo que valores negativos indicam que a proteína possui potencial de interação de natureza hidrofílica com água
**Estimated half-life**	10	

Table S3. Sequence ID of organisms belonging to the seven families of MCOs used to construct the phenetic tree

Organism	Superfamily	Sequence ID
Stenotrophomonas maltophilia	H	WP_012511889.1
Pseudomonas lini	H	WP_050683288.1
Ralstonia pickettii	H	WP_012435784.1
Xanthomonas sacchari	H	WP_043093362.1
Ralstonia solanacearum	H	WP_014618718.1
Lysinibacillus fusiformis	I	WP_025115145.1
Bacillus coagulans	I	WP_017553860.1
Porphyromonas asaccharolytica	I	WP_028929477.1
Bacillus pseudomycoides	I	WP_003195677.1
Alicyclobacillus pomorum	I	WP_026965086.1
Serratia rubidaea	J	WP_054307096.1
Exiguobacterium marinum	J	WP_214798326.1
Dickeya chrysanthemi	J	WP_027712201.1
Lactobacillus sanfranciscensis	J	WP_014081426.1
Enterobacter hormaechei	J	WP_017383764.1
Aurantimonas coralicida	L	WP_187392575.1
Labrenzia alexandrii	L	WP_008195945.1
Nitratireductor indicus	L	WP_009452578.1
Ruegeria conchae	L	WP_010439294.1
Vibrio diabolicus	L	WP_031780602.1
Streptomyces scabiei	K	WP_199924177.1
Streptomyces mutabilis	K	WP_191888100.1
Streptomyces monomycini	K	WP_030021766.1
Amycolatopsis marina	K	WP_177242603.1

Cellulosimicrobium aquatile	K	SIQ11895.1
Blastopirellula marina	N	WP_002651199.1
Phycisphaera mikurensis	N	WP_014437621.1
Rhizobium etli	N	WP_020921561.1
Methylotenera versatilis	N	WP_148218589.1
Derxia gummosa	N	WP_028312029.1
Chamaesiphon minutus	O	WP_015160154.1
Leptospira santarosai	O	WP_004462854.1
Bradyrhizobium elkanii	O	WP_028351214.1
Fictibacillus aquaticus	O	WP_094253906.1
Sinorhizobium fredii	O	WP_080603998.1

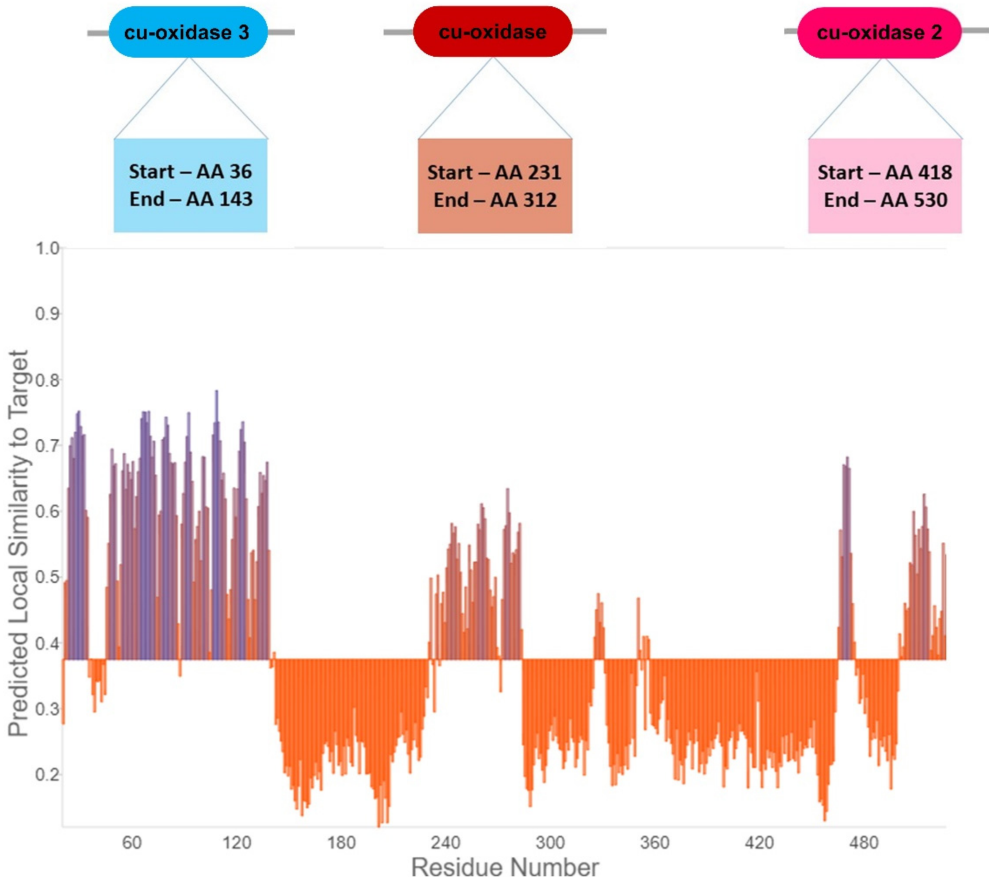

Figure S1. Predicted Local Similarity of Lac_CB10 demonstrating the best prediction in the conserved domains of cu-oxidase.

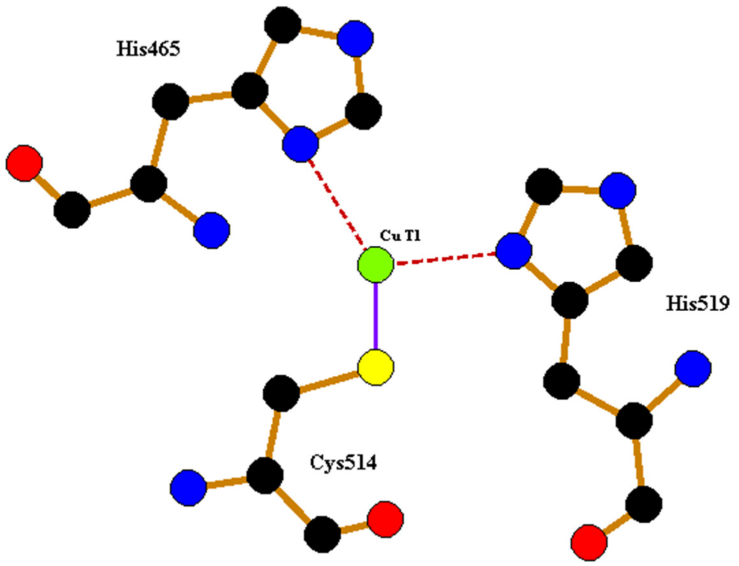

Figure S2. T1 copper center coordinated by a cysteine residue and two histidines schematized by LigPlot+ software version 2.2.4 [60]. Red dashed lines indicate hydrogen bonds; the connectors of the ligands are colored in purple; and amino acid residues are shown in orange.

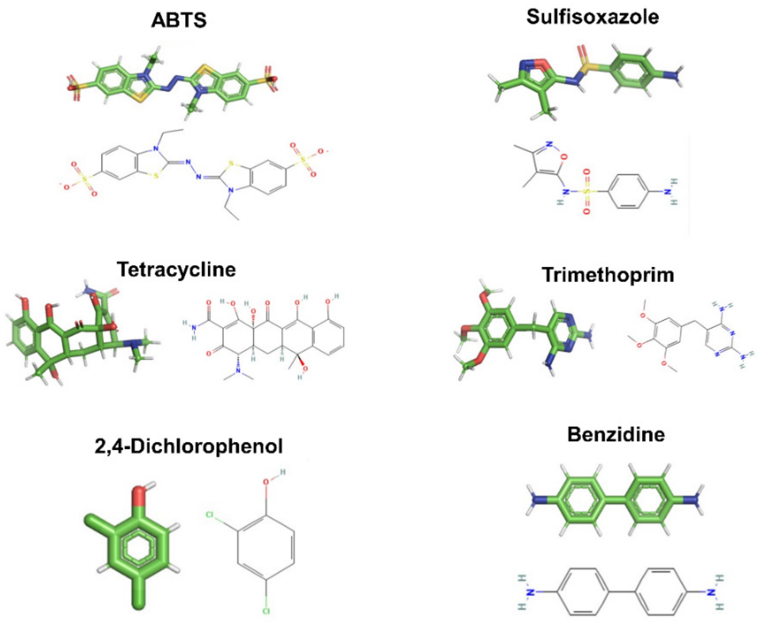

Figure S3. 2D and 3D structures of the ligands used in the molecular docking study with Lac_CB10.

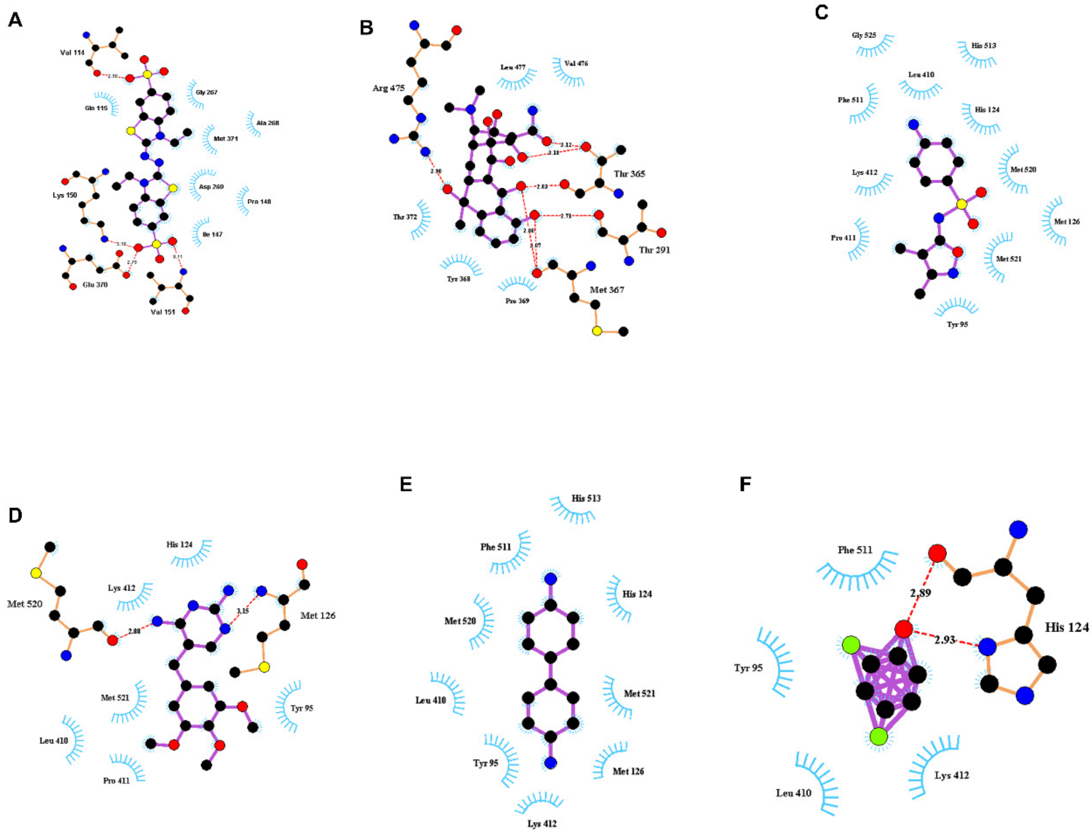

Figure S4. Hydrophobic interactions between ligant and Lac_CB10 schematized by LigPlot+ software version 2.2.4 [60]. Red dashed lines indicate hydrogen bonds with their length in angstroms; light blue icons indicate residues involved in hydrophobic contacts; the connectors of the ligands are colored in purple; and amino acid residues are shown in orange. A. Laccase-ABTS complex. B. Laccase-tetracycline complex. C. Laccase-sulfisoxazole complex. D. Laccase-trimethoprim complex. E. Laccase-benzidine complex. F. Laccase-2,4-DCP complex.

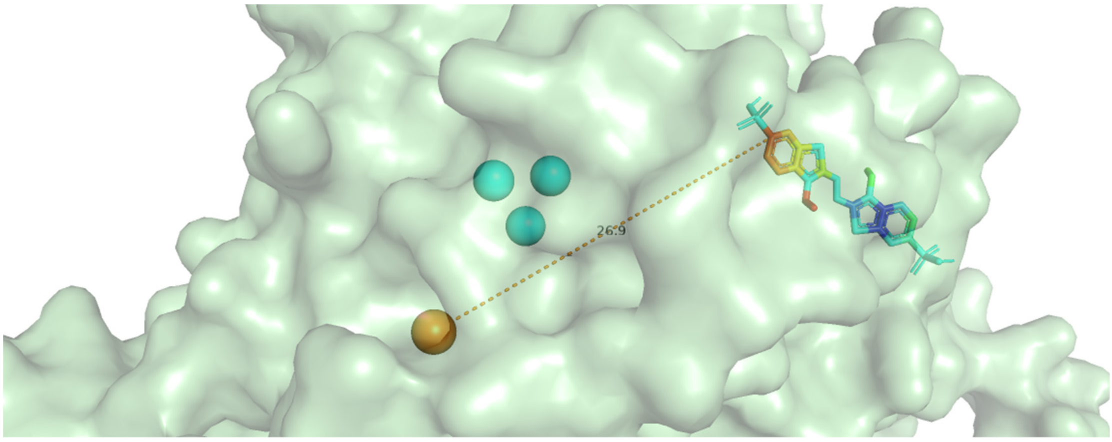

Figure S5. Three-dimensional image showing the distance of 26.9 Å between the ligand, represented by sticks, and the copper center T1, represented as an orange sphere.
